# Influence of Solvent Evaporation Temperature on the Performance of Ternary Solid Polymer Electrolytes Based on Poly(vinylidene fluoride-*co*-hexafluoropropylene) Combining an Ionic Liquid and a Zeolite

**DOI:** 10.1021/acsaem.3c00155

**Published:** 2023-05-08

**Authors:** João C. Barbosa, Daniela M. Correia, Arkaitz Fidalgo-Marijuan, Renato Gonçalves, Stanislav Ferdov, Verónica de Zea Bermudez, Carlos M. Costa, Senentxu Lanceros-Mendez

**Affiliations:** † Physics Centre of Minho and Porto Universities (CF-UM-UP) and Laboratory of Physics for Materials and Emergent Technologies, LapMET, University of Minho, 4710-057 Braga, Portugal; ‡ CQ-VR, University of Trás-os-Montes e Alto Douro, 5000-801 Vila Real, Portugal; § Center of Chemistry, University of Minho, 4710-057 Braga, Portugal; ∥ BCMaterials, Basque Center for Materials, Applications and Nanostructures, UPV/EHU Science Park, 48940 Leioa, Spain; ⊥ Department of Organic and Inorganic Chemistry, University of the Basque Country UPV/EHU, 48940 Leioa, Spain; # Department of Chemistry, University of Trás-os-Montes e Alto Douro, 5000-801 Vila Real, Portugal; ¶ Institute of Science and Innovation for Bio-Sustainability (IB-S), University of Minho, 4710-053 Braga, Portugal; ● Ikerbasque, Basque Foundation for Science, 48009 Bilbao, Spain

**Keywords:** ternary composites, PVDF-HFP, evaporation temperature, solid polymer electrolytes, lithium-ion batteries

## Abstract

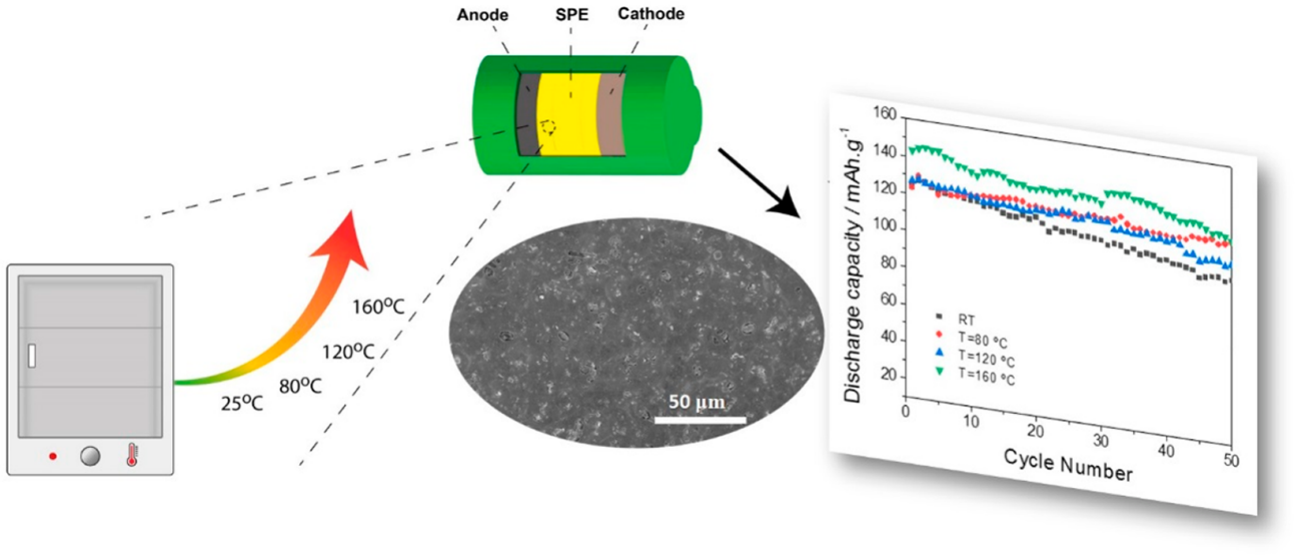

Solid polymer electrolytes (SPEs) will allow improving safety and durability in next-generation solid-state lithium-ion batteries (LIBs). Within the SPE class, ternary composites are a suitable approach as they provide high room-temperature ionic conductivity and excellent cycling and electrochemical stability. In this work, ternary SPEs based on poly(vinylidene fluoride-*co*-hexafluoropropylene) (PVDF-HFP) as a polymer host, clinoptilolite (CPT) zeolite, and 1-butyl-3-methylimidazolium thiocyanate ([Bmim][SCN])) ionic liquid (IL) as fillers were produced by solvent evaporation at different temperatures (room temperature, 80, 120, and 160 °C). Solvent evaporation temperature affects the morphology, degree of crystallinity, and mechanical properties of the samples as well as the ionic conductivity and lithium transference number. The highest ionic conductivity (1.2 × 10^–4^ S·cm^–1^) and lithium transference number (0.66) have been obtained for the SPE prepared at room temperature and 160 °C, respectively. Charge–discharge battery tests show the highest value of discharge capacity of 149 and 136 mAh·g^–1^ at C/10 and C/2 rates, respectively, for the SPE prepared at 160 °C. We conclude that the fine control of the solvent evaporation temperature during the preparation of the SPE allows us to optimize solid-state battery performance.

## 1 Introduction

As modern society demands electronic devices with higher efficiency and better performance, there is an increasing need for improved and safer energy storage devices. Currently, the most efficient devices available nowadays for this purpose are lithium-ion batteries (LIBs), being therefore widely used in a vast array of applications, ranging from small portable electronic devices to electric vehicles.^1^ LIBs are able to deliver a higher energy amount per unit of mass and volume when compared with the other options currently in the market. They show long lifecycles and no memory effects, making them the most suitable device for application in the scope of the fourth industrial revolution that we are facing.^2^ The main limitation of the currently employed LIBs is the use of liquid electrolytes in their structure, which due to their high toxicity and flammability holds back the safety and environmental impact of these systems.^3,4^

Solid electrolytes have been therefore under study in order to overcome this issue. The aim of these electrolytes is to replace the current separator/electrolyte systems by providing a solid material with high ionic conductivity while maintaining the electronic insulator function.^5^ Solid electrolytes can be divided into two distinct categories: inorganic electrolytes, typically composed of ceramic materials, and polymer electrolytes, which comprise a polymer matrix combined with different fillers.^6^

Solid polymer electrolytes (SPEs) have almost 50 years of history that were initiated with the first works on poly(ethylene) oxide (PEO) and lithium salts.^7^ Since then, the field of SPE investigation expanded significantly as research efforts were focused on improving the SPE room-temperature ionic conductivity and interfacial compatibility.^8^ To achieve this purpose, different materials were proposed. As far as the polymer matrix is concerned, there are reports on the use of PEO,^9^ poly(ethylene glycol) (PEG),^10^ poly(urethane),^11^ poly(acrylonitrile) (PAN),^12^ cellulose,^13^ and various poly(vinylidene fluoride) (PVDF) copolymers.^14^ In particular, it was recognized that the properties of poly(vinylidene fluoride-*co*-hexafluoropropylene) (PVDF-HFP), namely, its polar phases, low degree of crystallinity, and high dielectric constant,^12^ are very attractive in this context, leading to an increasing amount of research involving this polymer.^15−17^

The choice of the polymer is usually accompanied by the addition of one or several fillers, which can act directly on the improvement of the ionic conductivity (active fillers) or other relevant properties of the SPE, such as thermal and mechanical stability or interfacial compatibility (passive fillers).^8^

The most common active fillers are lithium salts, such as lithium bis(trifluoromethanesulfonyl)imide (LiTFSI)^18^ and lithium perchlorate (LiClO_4_).^19^ However, ionic liquids (ILs) have gained increasing relevance in the field in the past decade, due to their ability to reduce the crystallinity of the polymer, which adds to the direct increase in the ionic conductivity caused by their presence as well as their nonflammable nature and low toxicity.^20^ 1-Butyl-3-methylimidazolium thiocyanate ([Bmim][SCN]) is reported to be one of the most promising ILs for the development of SPEs based on PVDF-HFP, due to its high ionic conductivity and its ability to improve the PVDF-HFP polar β-phase content, which in turn increases Li^+^ dissociation and consequently the ionic conductivity.^16^

Regarding passive fillers, they allow us to improve the properties of the SPE, such as thermal and mechanical stability, contributing to further enhancing SPE operation.^8^ Although these passive fillers have usually been ceramics,^21^ in recent years the focus has also been placed on microporous materials, such as metal–organic frameworks (MOFs)^22^ and zeolites.^23,24^ The interest in this kind of materials is mainly due to their high surface area and porous structure which allow a large number of interactions with other fillers and polymers, resulting in improved stability.^8,24^ Different zeolites were studied recently, with clinoptilolite (CPT) being one of the most promising ones on account of its high thermal stability, low density, and high surface area.^25,26^ Its ion exchange capacity and the possibility to include lithium ions in the CPT structure proved to improve battery capacity through the increase in the number of charge carriers.^27^

Despite the importance of the materials selected in the SPE production, the preparation method is also a critical step to achieve optimized battery performance. In fact, it has been reported that the order of addition of the components has a significant influence on the battery stability with a capacity retention variation from 8 to 76% and a discharge capacity variation from ∼25 to 160.3 mAh·g^–1^ at a C/15-rate after 50 cycles, depending on the addition order of the fillers.^24^

In this scope, an important parameter that has not been yet examined in SPE development is the solvent evaporation temperature during the processing of three-component systems, despite its scientific and technological relevance. The effect of the solvent evaporation temperature on the electrochemical properties of gel polymer electrolytes has been addressed, nevertheless, for a binary system with poly(vinylidene fluoride) polymer and 1-butyl-3-methylimidazolium bis(trifluoromethylsufonyl)imide.^28^ It must be emphasized that in the case of a polymer/solvent solution the solvent evaporation rate, depending on the processing temperature, strongly affects sample morphology and its physical-chemical, thermal, and electrical properties,^29^ which in turn will determine battery performance. In addition, the filler proportion for these ternary SPEs with two synergetic fillers has been optimized.^24,27^ Thus, the goal of this work is to study the effect of solvent evaporation temperature during SPE processing by varying the temperature from room temperature to 160 °C, allowing both a deep understanding of the system and optimized performance. The ternary SPE studied is based on PVDF-HFP, CPT, and [Bmim][SCN], and the effect of the processing conditions on SPE morphology, physical-chemical characteristics (crystallinity and polar β-phase content), thermal and mechanical stability, ionic conductivity, electrochemical stability window, lithium transference number, and battery performance is reported.

## 2 Experimental Section

### 2.1 Materials

Poly(vinylidene fluoride-*co*-hexafluoropropylene) (PVDF-HFP) (Kynarflex PVDF-HFP 2801-00107) was obtained from Arkema. Clinoptilolite (CPT) and 1-butyl-3-methylimidazolium thiocyanate ([Bmim][SCN]) were supplied by Newstone International LLC, Japan, and by Iolitec, respectively. The solvents *N*,*N*-dimethylformamide (DMF, 99%) and *N*-methyl-2-pyrrolidone (NMP, 99%) were purchased from Merck. For the electrode preparation, PVDF (Kynar PVDF HSV900), lithium iron phosphate (LiFePO_4_ (LFP)), and Super P conductive carbon black were supplied by Arkema, Phostech Lithium, and Timcal Graphite & Carbon, respectively.

### 2.2 Sample Preparation

**Figure 1:**
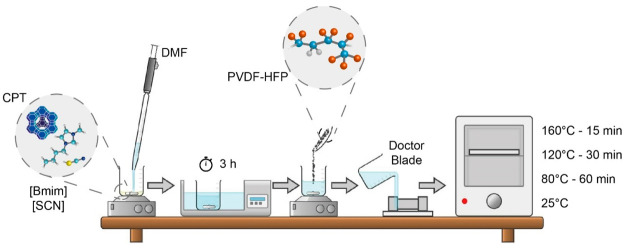
Schematic representation of the sample preparation process and conditions.

The doctor blade technique (Figure 1) was used for the preparation of the samples using a polymer/zeolite weight ratio of 84:16 and a polymer/IL weight ratio of 60:40, as these ratios are reported to be those that optimize functional properties without compromising the structural integrity of the samples and warranting mechanical characteristics, so that they do not present a gel behavior.^24^

The zeolite and the IL were mixed together, and then the DMF solvent was added. The solution was then dispersed for 3 h in an ultrasonic bath (ATU, model no. ATM40–3LCD). The PVDF-HFP polymer was added subsequently, and the resulting solution was placed under magnetic stirring (Ika, model no. C-MAG HS 7) for 30 min until complete dissolution of the polymer. The resulting solution was cast onto a glass substrate, and a doctor blade was used to uniformize the thickness to around 80 ± 5 μm. For the sample evaporated at room temperature, a thickness of 100 ± 5 μm was obtained due to the phase separation process and the porous microstructure. The samples were then placed in an oven (PSelecta) at different temperatures to evaporate the solvent at different rates; i.e., some of the samples were evaporated at room temperature (RT), whereas other samples were placed at 80 °C for 1 h (T80), 120 °C for 30 min (T120), and 160 °C for 15 min (T160). In order to guarantee the total solvent evaporation from the ternary composites, different times for the different solvent evaporation temperatures were used, taking into account previous works focused on the study of evaporation kinetics of similar systems.^30^

### 2.3 Sample Characterization

The morphology of the ternary SPEs was examined by scanning electron microscopy (SEM) at 10 kV with a Carl Zeiss AG-EVO 40 Series equipment. The samples were previously deposited with a conductive layer of gold by sputtering with a Polaron model SC502.

X-ray diffraction (XRD) patterns were conducted using a Panalytical X’pert Cu Kα diffractometer in the range 2θ = 5–70°, with a step size = 0.015° and exposure time of 10 s/step. The degree of crystallinity of each ternary SPE sample was obtained through the DIFFRAC.EVA (Bruker, AXS) software package, taking into consideration that they are semicrystalline.^31^ Thus, the amorphous phase content was calculated using eq 1:
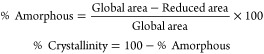
1The polymer phase was evaluated by Fourier transform infrared (FTIR) spectroscopy in the attenuated total reflection (ATR) mode using Jasco FT/IR-6100 equipment over a range from 600 to 4000 cm^–1^ at a resolution of 4 cm^–1^ with 64 scans. The polymer β-phase (*F*(β)) content of each ternary SPE was determined using eq 2(32)
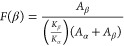
2where *A*_α_ and *A*_β_ are the absorbances at 760 and 840 cm^–1^, corresponding to the α and the β phases of the polymer, respectively. *K*_α_ and *K*_β_ are the absorption coefficients for these bands (6.1 × 10^4^ and 7.7 × 10^4^ cm^2^ mol^–1^, respectively^32^).

The thermal properties of the samples were evaluated using differential scanning calorimetry (DSC) and thermogravimetric analysis (TGA). DSC was carried out (PerkinElmer DSC 6000 instrument) under a nitrogen atmosphere in the temperature range between 20 and 200 °C at a heating rate of 10 °C min^–1^. TGA was performed using a NETZSCH STA 449F3 thermobalance under a nitrogen atmosphere between 20 and 800 °C at 5 °C min^–1^. Measurements were performed in a crucible comprising about 10 mg of weight for each sample.

Stress–strain mechanical measurements were carried out at room temperature and at a strain rate of 15 mm/s with a TST350 tensile testing stage from Linkam Scientific Instruments. The ternary SPE samples were previously prepared with dimensions of 30 mm × 10 mm × 50 μm.

The ionic conductivity (σ_i_) was obtained by electrochemical impedance spectroscopy using Autolab PGSTAT-12 (Eco Chemie) equipment in the temperature range from 25 to 80 °C, frequency range from 0.1 mHz to 106 Hz, and 10 mV of amplitude. Measurements were performed in Gold |SPE| Gold electrode (Goodfellow, >99.95% of 10 mm diameter) symmetry cells. The samples were pretreated at 60 °C in a Buchi TO51 tube oven with a type K thermocouple. The ionic conductivity (σ_i_) of the samples was determined using eq 3
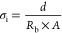
3where *d* is the thickness of the SPE sample, *R*_b_ is the bulk resistance; and *A* is the area.

The temperature (*T*) dependence of σ_i_ in the ternary SPE follows the Arrhenius equation (eq 4) in the measured range
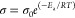
4where *E*_a_ is the apparent activation energy; *R* is the gas constant (8.314 J mol^–1^ K^–1^); and σ_0_ is a pre-exponential factor.

Cyclic voltammetry was conducted through a two-electrode cell configuration (25 μm diameter gold microelectrode |SPE| lithium metal) using an Autolab PGSTAT-12 (Eco Chemie) at 0.1 V s^–1^ between 0 and 4.5 V within a dry argon-filled glovebox at room temperature.

The Li-ion transference number (*t*_Li_^+^) was determined using symmetrical lithium cells by the potentiostatic polarization method by applying a DC voltage of 10 mV at room temperature. The *t*_Li_^+^*I* value was obtained through the Bruce and Evans equation (eq 5)^33^
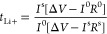
5where *I*_0_ is the initial current; *I*_s_ is the steady current; Δ*V* is the applied potential; and *R*^0^ and *R*^s^ are the initial and final resistances of the Li electrode/electrolyte before and after polarization, respectively.

### 2.4 Battery Testing

Cathodic half-cells (Li |SPE| LFP cathode) were assembled in a glovebox under an argon atmosphere (H_2_O, O_2_ < 1 ppm). The materials were dried overnight in a Buchi TO51 tube oven at 60 °C, under vacuum, before being transferred to the glovebox. LFP cathodes are composed of an active material/conductive material/polymer binder weight ratio of 80:10:10 with an active mass loading of ∼3.28 mg·cm^–2^, an area of 50.24 mm^2^, and a thickness of 35 ± 5 μm. More details about their preparation are given in ref (34).

Galvanostatic charge–discharge cycles were carried out in a Landt CT2001A instrument at C/10 rate (C = 170 mAh·g^–1^) for 50 cycles and at different rates (C/10, C/5, and C/2) for 10 cycles. The electrical properties of the assembled batteries were determined by impedance spectroscopy, using an Autolab PGSTAT12 instrument with a signal amplitude of 10 mV and a frequency range from 10 mHz to 500 kHz with open-circuit voltage between 3.2 and 3.4 V.

## 3 Results and Discussion

### 3.1 Morphology and Structural Properties

**Figure 2:**
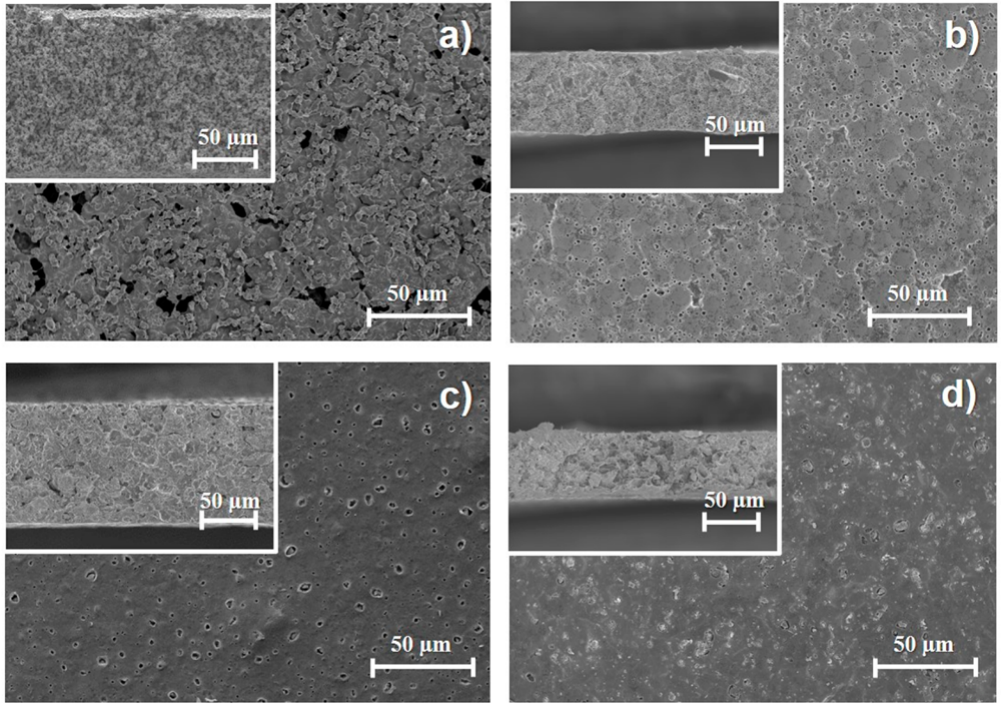
Surface and cross-section SEM images of the ternary SPE PVDF-HFP/CPT/[BMIM][SCN] samples prepared at RT (a), 80 °C (b), 120 °C (c), and 160 °C (d).

The morphology of the ternary SPEs prepared at different temperatures from RT to 160 °C is presented in the representative surface and cross-section SEM images of Figure 2. The surface SEM images of the samples demonstrate a good and homogeneous distribution of the fillers throughout the polymer matrix without the presence of large agglomerates, indicating a good compatibility between the different components.

The analysis of the SEM images shows that the processing temperature influences the morphology of the samples. The sample prepared at RT shows a porous texture due to a phase separation process^29^ and to the low evaporation temperature used, which reduces the solvent evaporation rate as well as the polymer chain mobility, limiting their capacity to occupy the free space left by the solvent.^35^ With increasing temperature and solvent evaporation rate, the phase separation process is reduced, and finally it is inhibited.^29^ The mobility of the polymer chains is increased, reducing the free space left by the solvent, which leads to a significant reduction in the samples’ porosity. This is particularly evident in the cross-section images in the insets of Figure 2. The remaining voids present at higher temperatures (120 and 160 °C) originate just from the presence of the CPT zeolite in the structure.^24^ PVDF-HFP spherulites are not evidenced in any sample due to the high amount of filler in the samples that limits the crystallization of the polymer.^36^

**Figure 3:**
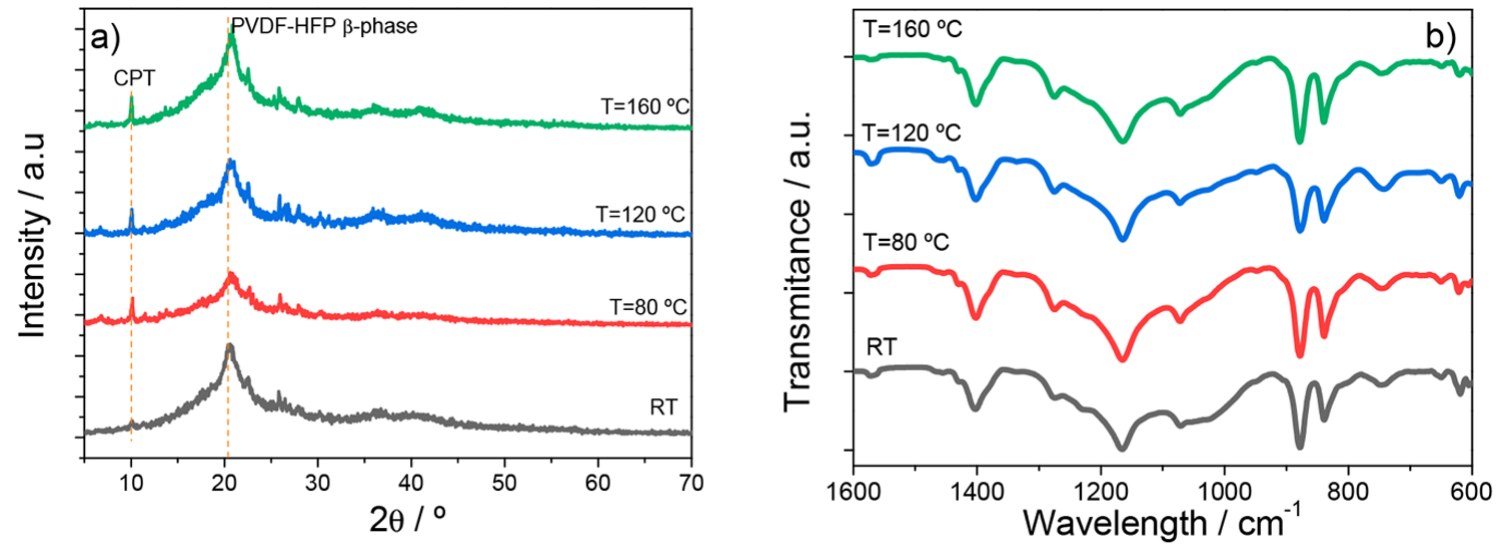
(a) XRD patterns and (b) ATR/FTIR spectra of the ternary SPE PVDF-HFP/CPT/[Bmim][SCN] samples.

Figure 3 shows the XRD patterns (Figure 3a) and ATR/FTIR spectra (Figure 3b) of the ternary SPE samples prepared at different temperatures. The characteristic crystalline peak of the zeolite observed at 10°^37^ (Figure 3a) confirms the presence of this filler. The intensity of this peak is independent of the solvent evaporation temperature. The peak at 20.26° that corresponds to the polar β phase of PVDF-HFP corresponds to the (110) (200) crystalline planes.^32^ The degree of crystallinity of the samples, determined through eq 1, is presented in Table 1. The samples with the lowest degrees of crystallinity are those prepared at RT and 160 °C due to the polymer crystallization governed by the phase separation dynamics^29^ in the former case and to the rapid solvent evaporation process at higher temperatures that leads to ill-crystallized regions^29^ in the latter case.

**Table 1:** Enthalpy, Degree of Crystallinity, β-Phase Content, Young Modulus, and Yield Stress for the Ternary Composites Prepared at Different Temperatures

Sample processing temperature/°C	Enthalpy/J·g^–1^ and degree of crystallinity/% ± 2%	β-phase/% ± 2%	Young modulus/MPa ± 2 MPa	Yield stress/MPa ± 0.1 MPa
RT	24.1/26.9	84	7	0.2
80	20.3/33.1	86	45	1.5
120	20.2/33.2	90	83	2.4
160	17.7/29.7	89	95	2.7

The effect of the processing temperature on the SPE chemical structure and polymer conformation was assessed by ATR/FTIR analysis (Figure 3b). The typical bands corresponding to the stretching vibrations of CH_2_ and CF_2_ of the PVF-HFP matrix are present in all the samples at 976, 795, 763, and 678 cm^–1^.^32^ The characteristic asymmetric stretching band of the Al–O bonds, attributed to the CPT zeolite, is also observed at 1087 cm^–1^.^38^ The high amount of [Bmim][SCN] IL in the samples leads to a dominant polymer chain conformation corresponding to the planar zigzag, which indicates a polar β-phase content above 80%, as demonstrated by the high intensity of the 840 cm^–1^ band. The β-phase content calculated for all samples is presented in Table 1. The different solvent evaporation temperatures used for sample preparation do not have a significant influence on the polymer conformation in the present case, as the main driver for the crystallization of the β-phase is the ion–dipole interaction between the IL and the polymer chains.^39^

### 3.2 Thermal and Mechanical analysis

**Figure 4:**
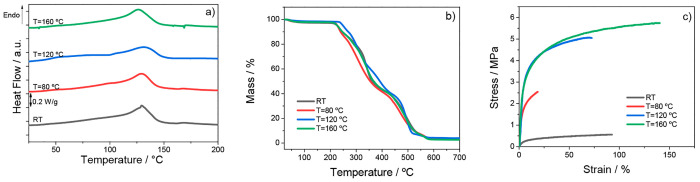
(a) DSC thermograms, (b) TGA curves, and (c) stress–strain characteristic curves of the ternary SPE PVDF-HFP/CPT/[Bmim][SCN] samples.

The influence of the presence of CPT zeolite and IL in the thermal and mechanical properties of the ternary SPEs samples prepared at different temperatures was evaluated by DSC and TGA, and the results are presented in Figures 4a and 4b, respectively.

DSC analysis allowed getting insight into the thermal behavior of the samples. The presence of the IL in the polymer matrix led to a destabilization of the SPE crystalline structure, resulting in a lower melting temperature than that reported for pristine PVDF-HFP (145 °C).^24^ This destabilization is attributed to the electrostatic ion–dipole interactions between the polymer matrix and the IL.^40^ The resulting melting temperature is around 125 °C, regardless of the sample’s production temperature, as shown in Figure 4a. The enthalpy area, related to the degree of crystallinity, is represented in Table 1, revealing a slight decrease with increasing processing temperature, in particular for higher processing temperature (160 °C), being in agreement with the results obtained from the XRD data (Figure 3a), suggesting a positive effect for battery performance since the ion conduction process occurs mainly through the amorphous part of the polymer.^41^

The thermal degradation behavior of the samples has been evaluated by TGA (Figure 4b). Distinct degradation steps associated with the different components of the samples are evident. The CPT degradation step occurs around 475 °C,^42^ thus overlapping with the PVDF-HFP degradation step at nearly the same temperature.^42^ The [Bmim][SCN] degradation occurs at lower temperatures (265 °C). However, in the prepared samples this process is shifted to higher temperatures due to the interaction between the IL and the CPT zeolite, as reported previously.^24^ These findings lead us to conclude that the processing temperature does not have a significant influence on the thermal degradation of the samples, as all of them present similar behaviors and degradation temperatures and steps.

Figure 4c reproduces the stress–strain characteristic curves of the prepared samples, providing information about their mechanical properties, which were evaluated by the parameters presented in Table 1. All the samples are characterized by the typical stress–strain behavior of a thermoplastic polymer composed of the elastic and plastic regimes separated by the yielding region.

The observed mechanical reinforcement effect of the CPT upon inclusion in a polymer matrix reported in previous works^24,27^ is attributed to the restriction of the polymer chain motion due to the presence of the zeolite, as demonstrated by the high Young modulus values obtained when compared with those of the pristine polymer (373 MPa).^43^ The exception is the sample prepared at the RT sample, which presents a low Young modulus because of its porous structure.^44^ We may thus infer that the effect of the IL, which typically leads to a plasticizing effect in the matrix,^39^ leading to a decrease of the Young modulus, is overcome by the presence of the zeolite. Regarding the different processing temperatures, it seems that higher temperatures lead to more rigid samples with a more compact microstructure, as proved by the increasing Young modulus and the elongation at break.

### 3.3 Ionic Conductivity, Electrochemical Window, and Lithium Transference Number

**Figure 5:**
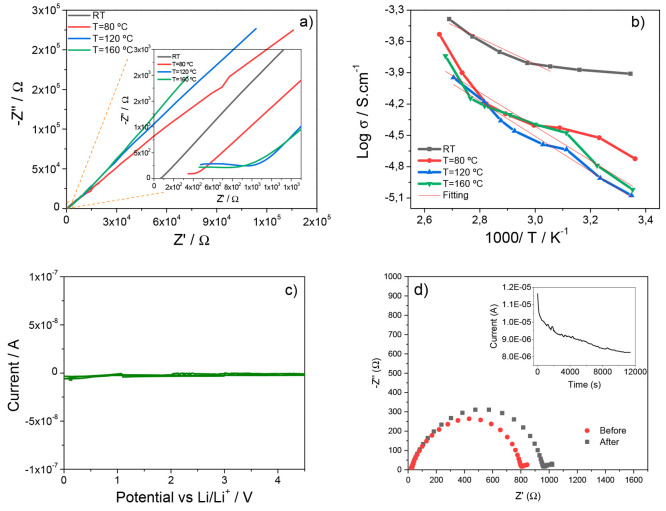
(a) Nyquist plots of the ternary SPE PVDF-HFP/CPT/[Bmim][SCN] samples. Inset: amplification of the Nyquist plot at high frequencies. (b) Arrhenius plots for the SPE PVDF-HFP/CPT/[Bmim][SCN] samples. (c) Cycle voltammogram and (d) lithium-transference number for the sample obtained at 160 °C. Inset: current vs time.

To assess the samples’ suitability when applications as SPE for LIBs are envisaged, electrochemical tests were carried out. Electrochemical impedance spectroscopy was used to evaluate the ionic conductivity of the samples. The obtained Nyquist plots are presented in Figure 5a.

The Nyquist plots are typically characterized by three characteristic regions which are a high frequency semicircle corresponding to the charge transfer process, a transition zone indicating the diffusion of counterions inside the electrode, and a line at lower frequencies associated with ion diffusion.^45^ The latter is the main process in the prepared samples as the presence of the IL significantly increases the number of mobile charge carriers.^46^ Furthermore, Figure 5a shows that the presence of the semicircle depends on the evaporation temperature, which could be attributed to the more compact structures obtained at higher temperatures.^29^ By analyzing the Nyquist plots at different temperatures, it is possible to determine the characteristics of the ionic conductivity through the Arrhenius equation (Figure 5b). The obtained plots show the typical increase in the ionic conductivity with increasing temperature attributed both to the increase of free charges resulting from the IL dissociation and to the increase in the mobility of the mobile ionic species and the polymer chains, together with the segmental relaxation of the polymer chains.^47^ In particular, A change in the slope of the ionic conductivity around 60 °C associated with the α-relaxation of the polymer is observed.^48^ The samples processed at RT are characterized by a less compact structure due to the phase separation process,^29^ which leads to the highest ionic conductivity among the prepared samples (up to 1.2 × 10^–4^ S cm^–1^ at RT). Furthermore, the higher ionic conductivity value of this sample is due to the low degree of crystallinity, as the ionic conductivity also depends on factors such as microstructure, crystallinity, and the related mechanical characteristics. The other samples show similar temperature behaviors, the main difference being in the value of the electrical conductivity, as shown in Table 2. Also, the activation energy value for all samples is low, with values below 14 kJ mol^–1^ demonstrating the low thermal energy required for the ion hopping process.

**Table 2:** Electrochemical Parameters of the Prepared Samples: Ionic Conductivity at Different Temperatures, Activation Energy, and Lithium Transference Number

	σ_i_/S·cm^–1^			
sample	25 °C	60 °C	activation energy/kJ mol^–1^	Li^+^ transference number
RT	1.2 × 10^–4^	1.6 × 10^–4^	11.9	0.51
*T* = 80 °C	1.9 × 10^–5^	3.2 × 10^–5^	9.9	0.35
*T* = 120 °C	8.5 × 10^–6^	3.5 × 10^–5^	12.8	0.64
*T* = 160 °C	9.5 × 10^–6^	3.7 × 10^–5^	14.0	0.66

Figure 5c shows the cycle voltammogram for the PVDF-HFP/CPT/[Bmim][SCN] sample obtained at 160 °C, as representative of the rest of the samples, for which the behavior is similar. The cyclic voltammogram was obtained between 0 V to +4.5 at 0.1 V s^–1^, and good electrochemical stability was observed, with no anodic and cathodic peaks at current values below 10^–9^ A, being suitable for battery applications. In addition, the sample preparation temperature did not affect the electrochemical stability of the samples.

Regarding the lithium transference number, Figure 5d shows the corresponding curves for its calculation for the PVDF-HFP/CPT/[Bmim][SCN] sample prepared at 160 °C. The values of the lithium transference number for the different samples are given in Table 2. These data reveal that this parameter is affected by the sample processing temperature, leading to larger values for the samples obtained at higher temperatures due to microstructural features, which improve the ion diffusion through the amorphous phase of the sample^41^ as well as through the ion solvation by the entangled polymer chains.^49^ This value is due to the fact that the interaction of the CPT particles with the PVDF-HFP polymer chains favors a more compact microstructure and to the presence of the IL, which allows us to improve the ionic conductivity, leading to a highest value of the lithium transference number of 0.66 for the SPE evaporated at 160 °C. The sample evaporated at RT possesses a distinct behavior due to its porous structure, leading to a transference number of about 0.51. Also, a correlation between the lithium transference number and the thermal activation energy of the samples seems to exist, as they vary in a similar way.

The analysis of the overall electrochemical results allows us to conclude that the prepared samples are suitable for application in LIBs, due to the combination of high RT ionic conductivity and Li^+^ transference number as well as excellent electrochemical stability.

### 3.4 Battery Performance

**Figure 6:**
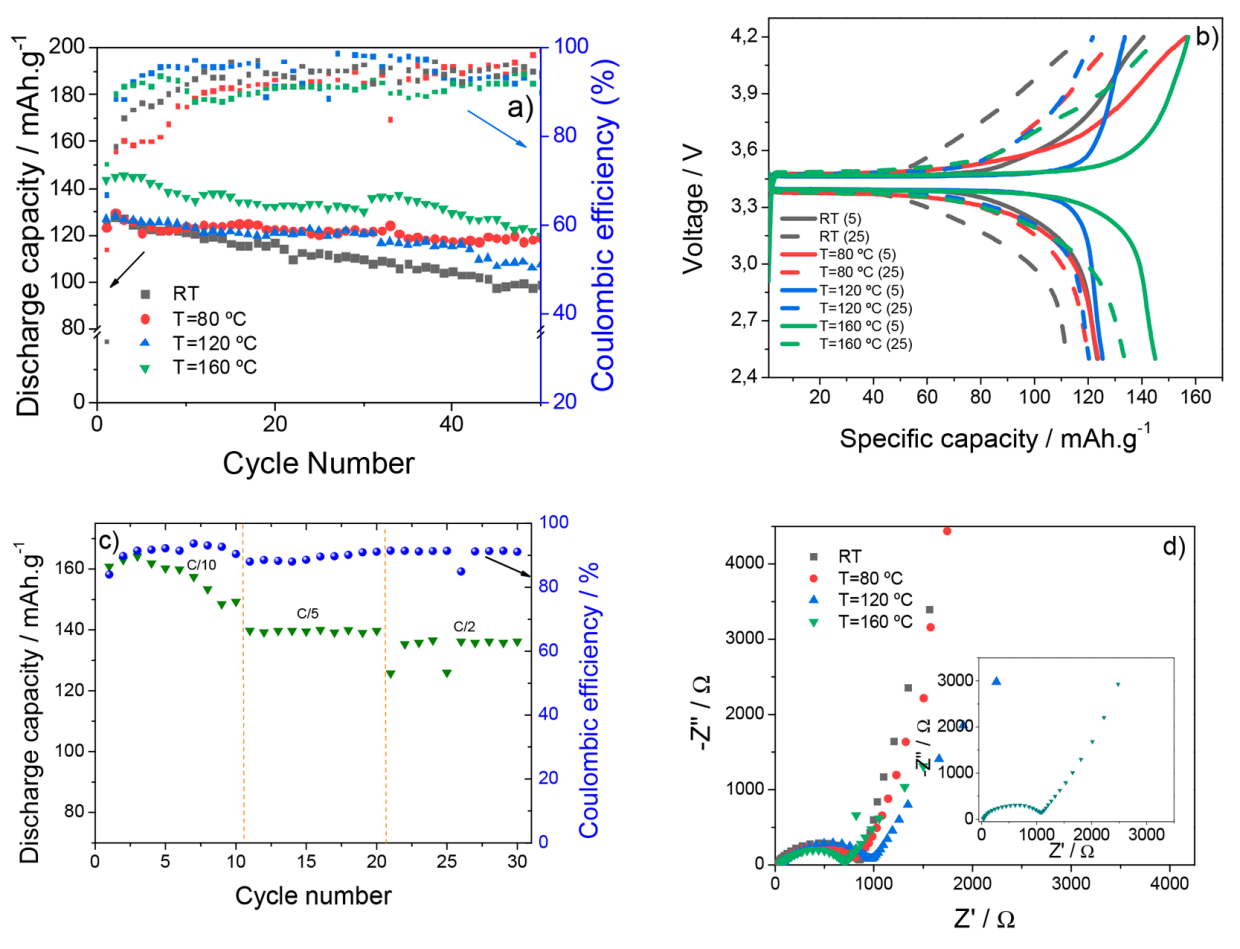
Cycling stability of the prepared samples at C/10 rate (a); charge/discharge profiles of the different samples at the 5th and 25th cycles at C/10 rate (b); rate performance of the SPE PVDF-HFP/CPT/[Bmim][SCN] sample prepared at 160 °C (c); and Nyquist plot of the assembled batteries before cycling (d) (inset: Nyquist plot after cycling for the sample prepared at 160 °C).

The prepared SPEs were assembled in LIBs, and their performance was evaluated through galvanostatic charge–discharge tests at room temperature and at C/10 rate. Both cycle life and rate performance tests were carried out at room temperature, to assess their suitability for solid-state battery applications. The results are presented in Figure 6.

The cycle stability tests (Figure 6a) show a high stability for all the prepared samples, with the highest discharge capacity value found for the SPE PVDF-HFP/CPT/[Bmim][SCN] prepared at 160 °C (145 mAh·g^–1^) and a capacity retention of 84% after 50 cycles. This behavior is attributed to the combination of the lower crystallinity of the sample and the high Li^+^ transference number, when compared to the other samples, such as the sample obtained at RT that exhibits a high ionic conductivity value. Some instability on the first cycles is attributed to the necessity to fully activate the system before the device is operational.^50^ This instability is also proven by the lower Coulombic efficiency value at the first cycles, which then stabilizes to about 80 to 100% efficiency. Despite the lower initial discharge capacity of the SPE sample obtained at 80 °C (123 mAh·g^–1^), its stability is significantly higher, being able to preserve 94% of its initial capacity after 50 cycles. This is proven by the charge–discharge profiles presented in Figure 6b, which show a small decay in the discharge capacity of the sample evaporated at 80 °C, despite the lower Coulombic efficiency when compared to other samples. The charge–discharge profiles also show the typical voltage plateau of the LFP active material between 3.3 and 4.5 V, representing the mechanism of insertion and extraction of the Li ions in the electrode’s structure.^51^ Considering that the best cycle life test is observed for SPE PVDF-HFP/CPT/[Bmim][SCN] obtained at 160 °C due to its high lithium transference number, Figure 6c shows the rate performance for this sample, presenting 10 cycles for each rate. The discharge capacity values for this sample were 149, 140, and 136 mAh·g^–1^ at C/10, C/5, and C/2 rates, respectively, and the discharge capacity value decreased with increasing C-rate due to the ohmic polarization effect.^52^ Except for the C/10 rate, the discharge capacity value is very stable for all cycle numbers as the sample shows high lithium transference number.

Impedance spectroscopy tests were carried out on the batteries, with the Nyquist plot of the samples before cycling being shown in Figure 6d. The three regions described above are present in these plots, with a bigger relevance of the semicircle at high frequencies, which is an indicator of the internal resistance of the battery components. Before cycling, a difference in overall resistance is observed between the different samples due to the variations in microstructure and surface compatibilization with the electrodes (Table 3). Also, there is an increase in the overall resistance of the batteries after cycling, which is ascribed to the formation of the solid electrolyte interphase (Table 3), the values, below 3694 Ω, being low due to the good compatibilization between the prepared samples and the electrode material.^53^ It is evidenced that the SPE solvent evaporation temperature influences this resistance, showing values ranging from 1067 to 3694 Ω, which decrease with increasing solvent evaporation temperature, both before and after cycling. This fact is also related to the better battery performance of the SPE sample prepared at 160 °C. Notice that, after cycling, the SPE samples prepared at 80 and 120 °C show the higher increase in resistance, which is attributed to a decrease of the compatibility with the Li metal electrode, due to the higher degree of crystallinity.

**Table 3:** Internal Resistance of the Assembled Batteries before and after Cycling

Sample	Resistance before (Ω)	Resistance after (Ω)
RT	877	1221
80	788	2547
120	866	3694
160	704	1067

The obtained results prove the suitability of this ternary composite system when compared to SPEs reported in the literature, as shown in Table 4.

**Table 4:** Literature Results for Related SPEs

Polymer	Component doping agents on dopants	Conductivity (S·cm^–1^**)**	Li^**+**^ transference number	Battery capacity of LFP batteries (mAh·g^**–1**^)/C rate	ref
PVDF-HFP	IL@UiO-67	4.3 × 10^–4^ (25 °C)	0.45	118 (1C); 25 °C	(54)
PVDF-HFP	LiTFSI, LLZTO	8.80 × 10^–5^ (25 °C)	0.27	158.7 (C/10); 25 °C	(55)
PVDF	LiClO_4_, LLTO	5.8 × 10^–4^ (25 °C)	0.80	152 (C/5); 25 °C	(56)
PEO	SSZ-13, LiTFSI	5.34 × 10^–2^ (70 °C)	0.85	154 (C/10); 60 °C	(23)
PEO	ZYNa zeolite, LiTFSI	1.66 × 10^–2^ (60 °C)	0.84	152 (C/5); 60 °C	(57)
PVDF-HFP	CPT, [Bmim][SCN]	1.2 × 10^–5^ (30 °C)	0.66	132 (C/2) 25 °C	This work

The obtained values are in line with those reported in the literature, which hints at the suitability of this approach for future developments in the solid-state battery field. The assembled batteries showed suitable ionic conductivity, a high Li^+^ transference number, and an excellent cycling stability with high discharge capacity values even at room temperature. Furthermore, the influence of the preparation method, namely, the processing temperature, is also stated, with a positive effect for higher sample preparation temperatures. This work highlights the importance of the solvent evaporation temperature as an important parameter on the preparation of SPEs. In particular, a correlation is observed between the degree of crystallinity, lithium transference number, and consequently battery performance, which proves the relevance of this parameter for further application at an industrial scale.

## 4 Conclusion

Ternary solid polymer electrolytes, SPEs, based on poly(vinylidene fluoride-*co*-hexafluoropropylene) (PVDF-HFP) as a polymer host, clinoptilolite (CPT) zeolite, and the ionic liquid (IL) (1-butyl-3-methylimidazolium thiocyanate ([Bmim][SCN])) as fillers were produced by a doctor blade technique, with varying solvent evaporation temperature, from RT to 160 °C. The effect of solvent evaporation temperature on the SPE morphology and thermal, mechanical, and electrical properties was analyzed. The microstructure of the SPEs is affected by the solvent evaporation temperature. Processing at RT leads to a porous morphology, whereas the samples prepared at higher temperatures are characterized by a compact morphology. Regardless of the processing temperature, excellent compatibility is observed between the zeolite, the IL, and the polymer matrix. The processing temperature slightly affected the degree of crystallinity of the samples, the melting and thermal degradation temperatures being practically independent of the processing conditions. At RT, the highest ionic conductivity was obtained for the sample obtained at RT (1.2 × 10^–4^ S cm^–1^), whereas the highest value of the lithium transference number (0.66) was obtained for the sample prepared at 160 °C. The charge–discharge behavior at RT for the sample processed at 160 °C shows excellent battery performance with 149 and 136 mAh·g^–1^ at C/10 and C/2 rates, respectively, which is attributed to the combination of two synergetic effects: ionic conductivity and lithium transference number. This work demonstrates that the processing temperature of the SPEs affects the battery performance due to its influence on sample morphology and physical-chemical properties, being a relevant parameter to consider in order to enhance the performance of RT solid-state batteries.
